# The use of virtual reality as a perspective-taking manipulation to improve self-awareness in Alzheimer’s disease

**DOI:** 10.3389/fnagi.2024.1376413

**Published:** 2024-04-25

**Authors:** Sofia Latgé-Tovar, Elodie Bertrand, Pascale Piolino, Daniel C. Mograbi

**Affiliations:** ^1^Institute of Psychiatry - Center for Alzheimer’s Disease, Federal University of Rio de Janeiro, Rio de Janeiro, Brazil; ^2^Laboratoire Mémoire, Cerveau et Cognition (LMC^2^), Institut de Psychologie, Université Paris Cité, Paris, France; ^3^Department of Psychology, Pontifical Catholic University of Rio de Janeiro, Rio de Janeiro, Brazil; ^4^Institute of Psychiatry – Psychology and Neuroscience King’s College London, London, United Kingdom

**Keywords:** Alzheimer’s disease, anosognosia, perspective-taking, self-awareness, virtual reality

## Abstract

Lack of awareness of symptoms or having a condition referred to as anosognosia is a common feature of individuals with Alzheimer’s Disease (AD). Previous literature on AD reported difficulties in evaluating self-abilities, often showing underestimation of limitations. There is increasing evidence that the perspective through which information is presented may moderate the performance appraisal and that anosognosia in AD might be a consequence of a deficit in assuming a third-person perspective. In this context, some studies showed that subjects may better recognize self-and other-difficulties when exposed to a third-person perspective. Considering the variety of approaches aiming to investigate the lack of awareness, there is still a scarcity of methods that provide great ecological validity and consider more than one facet of awareness, thus failing to offer more accurate evaluations of daily experiences. The present paper primarily addresses the theme of the multidimensional character of awareness of abilities in AD and the effect of perspective-taking on its trajectories. The focus turns to virtual reality as a promising tool for a greater evaluation of perspective-taking and self-awareness. Particularly, these systems offer the possibility to involve users in cognitive and sensorimotor tasks that simulate daily life conditions within immersive and realistic environments, and a great sense of embodiment. We propose that virtual reality might allow a great level of complexity, veracity, and safety that is needed for individuals with AD to behave according to their actual abilities and enable to explore the liaison between the subject’s viewpoint, performance, and self-evaluation. In addition, we suggest promising clinical implications of virtual reality-based methods for individualized assessments, investigating specific impacts on subjects’ life and possible improvements in their awareness.

## 1 Introduction

Self-awareness can be defined as the ability to take oneself as the object of awareness (Morin, 2011). It is a multidimensional construct, encompassing different sources of information (Mograbi et al., 2023) and it has been shown to be altered in people with dementia (PwD) (Mograbi et al., 2021). Specifically, lack of awareness of symptoms, cognitive loss, or having a condition, often referred to as anosognosia (Mograbi and Morris, 2018), is a common feature of different neurological disorders, including dementia (Morris and Hannesdottir, 2004; Mograbi et al., 2012b; Antoine et al., 2019). For instance, the frequency of unawareness of memory impairment can be greater than 60% in people with Alzheimer’s disease (PwAD) (Aalten et al., 2006; Mograbi et al., 2012b), but its association with dementia severity has recently been described as multifactorial and highly dependent on the inter-and intra-individual variability of profiles and trajectories of awareness (Mayelle et al., 2020).

Reduced awareness regarding dementia symptoms may be linked to an underestimation of limitations in AD (Spalletta et al., 2012; Wilson et al., 2016; Cacciamani et al., 2021), affecting treatment compliance (Patel and Prince, 2001; Sunderaraman and Cosentino, 2017), and predicting risk for hazardous behaviors (Alexander et al., 2021). Anosognosia reduces the quality of life in AD and contributes to caregiver burden (Mograbi et al., 2012a,b; Antoine et al., 2019).

Previous literature on AD reported difficulties in the evaluation of self-abilities (Brandt et al., 2018; Cacciamani et al., 2021), often showing overestimation of capacity (Duke et al., 2002; Souchay, 2007), but with evidence also pointing to underestimation of ability (Mograbi et al., 2012b). The latter suggests that difficulties in self-appraisal do not have a positive bias, potentially being better explained by neurocognitive factors. In any case, a variety of factors may impact the appraisal of self-ability, such as autobiographical memory disorder, behavioral alterations, emotional distress, attentional deficit, and defensive denial (Agnew and Morris, 1998; Tagai et al., 2020). There is growing evidence that the focus of the assessment and the perspective through which information is presented may modulate the appraisal of ability.

Using prediction and postdiction of performance in a word-list learning task, Duke et al. (2002) indicated that PwAD overestimate self-performances and underestimate their difficulties. Still, participants only overestimated their performances relative to their estimates of caregivers’ performance, but not relative to those by fictional characters. Mograbi et al. (2014) indicated that PwAD predicted others performed as well as them, arguing that a potential deficit to put themselves in someone else’s position could indeed be associated with a discrepancy in self-and others-perceiving. Clare et al. (2012a) have shown that when exposed to the performance of others through vignettes, PwAD accurately perceived deficits in another individual that were similar to theirs. This suggests that observation of others may be advantageous for evaluating and judging the self (Gambini et al., 2004; Marcel et al., 2004). These findings also suggest that knowledge about the self and knowledge about others might be represented through different processes.

There have been a variety of approaches developed to assess and investigate the lack of awareness of a condition and/or deficits and their particularities according to the object of awareness. Duke and colleagues suggested two main classes these tools would fall into, described as *performance prediction–postdiction* and *questionnaire discrepancy*. The first class is based on patient’s estimations before one’s own performance and one’s spouse or others or hypothetical characters (prediction) in relation to the estimation immediately after the completion of the related task (postdiction). The comparison between them results in a comparative prediction–postdiction accuracy index, which allows assessing the patient’s ability to adjust their evaluation about themselves and others, and increase the accuracy of judgments after the task. By way of example, the Multidimensional Isomorphic Simple Awareness Assessment is a prediction-performance discrepancy instrument described in Antoine et al. (2013), which contrast self-rating scores with actual performances on the Dementia Rating Scale, as well as the Cognitive Failures Questionnaire reported in Feehan et al. (1991), a self-rating score compared with the performance on multiple cognitive tests, and the Perceived Performance Questionnaire, reported in Graham et al. (2005), employed to contrast how well patients think they performed compared to others, and their actual cognitive impairment measure with the ADAS-cog (for a review of prediction-performance discrepancy instruments, de Ruijter et al., 2020). The *questionnaire discrepancy* relies into patient and caregiver reports over patients’ level of functioning, focusing on possible discrepancies between these two reports. The authors discuss how these methods encompass distinct vertices of consciousness, with pre-postdiction comprising tracking ongoing memory and prior knowledge of self and others, as well as more online monitoring of skills, while questionnaire discrepancies comprising broader memories beliefs of themselves (for a review, Duke et al., 2002).

In addition to the *patient–caregiver discrepancies* (questionnaire-based) and the p*rediction of performance discrepancies* (with a score of the level of anosognosia), de Ruijter et al. (2020) also suggested the *clinician rating* as a quantitative method to measure awareness. This tool would be based on interviews with the person or an informant, and on case records, allowing a simple and quick assessment and the analysis of a variety of objects of awareness, yet they are limited in the clinician’s interpretation of subjective answers and in investigating the domain-related variations in awareness due to the use of global ratings. The authors also discussed two recent methods for in-depth assessment, described as (1) Phenomenological methods, relying on verbal self-report and exploring the dynamic construction of awareness, through triangulation between participant and informant accounts; and (2) Multidimensional method, based on a combination of participant-carer and prediction-performance discrepancy measures, through clinician ratings of awareness, metamemory measures, and rating of a fictional patient presented on video (de Ruijter et al., 2020; for more reviews on the assessment methodologies, Duke et al., 2002; Howorth and Saper, 2003; Clare et al., 2005; Mayelle et al., 2019; Alexander et al., 2021).

Still, these methods rely on verbal self-report, highly compromised in dementia (Clare et al., 2005). For the most part, they lack ecological validity and fail to mimic daily life experiences, preventing individuals from behaving as in real life (Schultheis et al., 2002; Farias et al., 2003; for a review, Plancher et al., 2010). The ecological validity of neuropsychological tests is a highly discussed topic in cognitive neuroscience and it has been developed considering notions of veridicality (i.e., test performance must predict some characteristics of daily functioning), and verisimilitude [i.e., experimental requirements are similar to the actual needs of a patient’s activities of daily living (Franzen and Wilhelm, 1996; Parsons, 2015)]. The concept focuses on creating testing conditions mimicking real-life activities, affording evidence on individual functioning and performance which is capable to represent functionality in everyday life (Parsons, 2015; Muratore et al., 2019; Rizzo et al., 2020). For example, the Multitasking in the City Test (Jovanovski et al., 2012) encompasses a virtual city filled with environments (e.g., restaurants, grocery store, doctor’s office, and bank) that require participants to engage in self-monitoring, multitasking, planning, and decision-making skills as in real-world environments. This VR task assesses participants’ cognitive constructions, with references to everyday life and with a less structured design as with other implemented tasks (Jovanovski et al., 2012; Parsons, 2015) as opposed to the structure of a logical memory test (listening to a story and retelling it).

The absence of standard behavioral and objective measurements that consider more than one facet of awareness leads to non-individualized and inaccurate evaluation of an individual’s abilities (Sunderaraman and Cosentino, 2017; Muratore et al., 2019; Mayelle et al., 2020). This places virtual reality (VR) as a promising tool to manipulate perspective-taking and evaluate self-awareness of abilities in PwAD within highly realistic environments (Plancher et al., 2010; Muratore et al., 2019).

This three-dimensional computer-based technique enables humans to immerse themselves in and interact in real-time with artificial highly realistic settings that stimulate distinct senses, as well as cognitive and sensorimotor skills (Fuchs et al., 2006; Plancher et al., 2010). A high degree of immersion and interaction in a first-person perspective (1PP) via a head-mounted display creates a sense of presence and verisimilitude with real behavior (see, for example, Armougum et al. (2019)]. Through a process of ownership of a virtual body (e.g., visuo-motor synchrony with an avatar), VR produces a sense of bodily self-awareness that enhances virtual presence (Kilteni et al., 2012; Gorisse et al., 2017; Kim et al., 2019; Penaud et al., 2022). The veracity of VR techniques (Salamin et al., 2010; Plancher et al., 2012; Debarba et al., 2015), along with their safety (García-Betances et al., 2015), has facilitated the development of improved tools for assessment and remediation in PwAD (Serino et al., 2015; Plancher and Piolino, 2017; La Corte et al., 2019). Therefore, immersive VR techniques with a great level of spatial or physical presence could be an innovative approach to investigate the liaison between an individual’s viewpoint, performance, and self-evaluation in the context of the disease. A better understanding of the effects of perspective-taking on awareness of condition, symptoms or cognitive ability could lead to more effective and individualized care practices, preserving patients’ abilities and retaining self-identity. Nevertheless, only a few studies have fully explored the topic through highly ecological techniques such as VR (García-Betances et al., 2015; Muratore et al., 2019).

Considering this, in the current narrative review we will explore why and how VR is a relevant and reliable instrument for studies regarding the association between anosognosia and perspective-taking in AD. This study aims to bring together and integrate topics, such as the theoretical framework of anosognosia and perspective-taking in AD, methods and instruments available to assess awareness in PwAD, and new technological methods based on VRIn this manner, it proposes to identify common issues and describe new theoretical frameworks and perspectives for further research and clinical practices, in a narrative approach. It does not intend to focus particularly on each of these topics or to formulate a well-defined research question, therefore it does not fit into a systematic review approach. This latter form of review follows the primary intention of finding empirical evidence to answer a specific research question through methodical analysis, well-established inclusion criteria and a restrictive framework, which prevents a broader exploration as the one aimed for this review. For the main parts of the argument, the literature searches were conducted in PubMed, PsyncInfo, Scopus and Web of Science, with references of selected articles being checked for additional relevant articles. In Section 2, we discuss the concept of anosognosia and its’ manifestation in AD. Section 3 examines self-reflection and perspective-taking processes, and how could these be related to anosognosia in AD. Section 4 summarizes the availability of methods used to assess awareness of ability, symptoms and condition in PwD and methodological challenges of existing approaches. Section 5 describes the usage and the adaptability of VR tool. In Section 6 we propose that on account of its ability to develop a highly immersive environment and realistic setting, VR would allow to investigate phenomena such as self-evaluation, perspective-taking, and its potential influence on self-awareness. We will then conclude with a section of concluding remarks.

## 2 Anosognosia in AD

Anosognosia is considered a complex and heterogeneous concept, which varies within the presentation and severity of AD (Agnew and Morris, 1998). A variety of studies have shown that unawareness may change according to its object (e.g., memory deficits, changes in activities of daily living (ADL), communication, and the diagnosis itself) (Marcel et al., 2004; Clare et al., 2005; Mayelle et al., 2020), not being uncommon for individuals to acknowledge difficulties in one sphere but not in another (Kotler-Cope and Camp, 1995). In PwAD, anosognosia might behave in a domain-specific manner, with literature suggesting more impaired awareness of cognitive difficulties than of behavioral ones, with awareness of memory deficits as the most commonly impaired within the cognitive domain (Green et al., 1993; Kotler-Cope and Camp, 1995). Moreover, awareness has been conceptualized as a phenomenon that can be approached through distinct explanatory levels (Mograbi et al., 2014). At a higher-order level, psychological, social, and cultural factors, such as personality traits, language, and collective values are likely to modulate awareness and influence knowledge and acceptance of having a condition (Clare, 2004; Mograbi et al., 2012a,b; Mograbi et al., 2014). Also, higher-order processes, related to more complex information (e.g., attributions and expectations), can influence cognitive functions (e.g., memory and perception) and have an effect on the fluency of information from lower levels (Mograbi et al., 2014).

Within the neurocognitive approaches, the Cognitive Awareness Model (CAM) considers anosognosia a heterogeneous phenomenon, with primary, executive, and mnemonic forms, and explores it through specific cognitive modules (Agnew and Morris, 1998; Morris and Hannesdottir, 2004; Morris and Mograbi, 2013). The model suggests the existence of “comparator mechanisms” (Cm), responsible for monitoring the incoming episodic autobiographical experience and for continuously comparing it to the existing semantic personal knowledge, stored in a personal database (PDB). In the case of an error, the Cm would detect a mismatch between current and expected performance, with the PDB being updated and new somaticized representations of personal abilities being directed into conscious awareness.

There are three main forms of anosognosia according to this framework. In *executive anosognosia*, there is a fault in the Cm, with no further indication that the failure experienced is atypical. In that way, the PDB is not updated and faulty information is transferred to the Metacognitive Awareness System (MAS), possibly leading to a lack of awareness of deficits. In the case of *mnemonic anosognosia*, a mismatch would be detected through Cm but fail to be encoded (Agnew and Morris, 1998; Morris and Hannesdottir, 2004; Morris and Mograbi, 2013). The inefficiency in registering new episodic experiences would possibly culminate in erroneous references based on consolidated semantic knowledge that are more temporally graded in AD (for a review, Westmacott et al., 2004). Notably, semantic autobiographical memories and personal knowledge are better preserved than episodic autobiographical memories in AD (e.g., Piolino et al., 2003; Eustache et al., 2004; Martinelli et al., 2013; Lalanne et al., 2015). According to the reformulated CAM (Morris and Mograbi, 2013), *primary anosognosia* would be characterized by an impairment in the MAS. Conforming to, the MAS would be a higher-order emergent process, receiving inputs from the Cm regarding failure and successful performances, and the PDB. In cases with a mismatch between current and previous experiences, the error detection would reflect in updating the PDB and into awareness in the MAS. Notably, through an implicit mechanism, the Cm could be used as a way to conduct emotional and behavioral responses without enabling conscious awareness of related experiences (Morris and Mograbi, 2013).

The CAM also proposes different memory systems for self-and other-information processing (Agnew and Morris, 1998). The PDB is grounded on the Autobiographical Conceptual Memory System, which mainly contains the material basis for personal appraisal, with semanticized self-representations and lifetime knowledge, such as previous experiences related to work, personal relationships, and self-abilities. Both systems would store information concerning the self and would be integrated with other aspects of higher-level cognitive function linked to individual, social, and cultural experiences. On the other hand, the Generic Memory System, through the store of general semantic knowledge, functions as a foundation for the representation and evaluation of others’ traits and abilities. In this manner, the particularities of each process would perhaps allow people to differently acknowledge personal information through an external perspective, and conceivably, in the case of unaware individuals, maybe to better perceive their impairments (Morris and Mograbi, 2013).

## 3 Self-evaluation and perspective-taking in AD

Throughout self-reflection processes, looking at ourselves through a third-person perspective (3PP) imagery may be especially advantageous for the evaluation and judgment of the self (Gambini et al., 2004; Marcel et al., 2004). As a result, in a way of optimizing processes of self-appreciation, this reference imagery could spontaneously originate in processes of appraisal of the self (McCarroll, 2017). The 3PP, as compared to the 1PP, is a constructive process of inferring, which may be related to the Theory of Mind (ToM), (Ruby and Decety, 2001, 2003, 2004; D’Argembeau et al., 2007; Ruby et al., 2009). ToM is described as a component of self-awareness, related to the capacity to attribute mental states to others and appreciate what they might perceive, feel and know (Goldman, 2012). The relationship between ToM and self-awareness might be linked to the notion that for individuals to understand and infer the mental states of others, they need to appreciate and achieve a full representation of their own mental states and the impacts of their behavior (Lage et al., 2022). The 1PP has been described as the default mode for information processing (Vogeley et al., 2004), creating a “sense of self” through the protagonist of daily events (Damasio, 2003; Lage et al., 2022) and using self-reference to acquire outputs from external settings (Serino et al., 2014). Mograbi et al. (2009) indeed discussed how changes in perspective-taking and the capacity to put oneself in another person’s position could be a critical cognitive ability involved in the formation of the self.

In line with this, anosognosia in AD was already suggested to be partly derived from a compromised metabolism in networks involving self-referential processes and perspective-taking (Salmon et al., 2005), presenting possible alterations in both 1PP and 3PP (Bond et al., 2016; Demichelis et al., 2020). Ruby et al. (2009) discussed how possible impairments in cerebral network subserving perspective-taking would likely prevent PwAD from using the 3PP to assess self-and other-personality as a way to counterbalance their memory loss (Ruby et al., 2009). Consequently, subjects mostly rely on familiarity-based information and non-updated semantic representations (Klein et al., 2003). Also, deficits in egocentric representations were previously reported in PwAD and suggested as a consequence of the reduced capacity to construct allocentric maps, translate them for encoding, and later store them in the hippocampus (for a review, see Serino et al., 2014). Alterations in the hippocampus responses have been already associated with diminished subjective characteristics of memories encoded and retrieved through 3PP experiences versus 1PP (Piolino et al., 2008, 2009; Bergouignan et al., 2014).

Studies in AD have suggested that individuals might assess more accurately their own cognitive abilities (Clare et al., 2012a) or even others’ abilities when these are exposed through a 3PP (McGlynn and Kaszniak, 1991). In relation to this, some authors have discussed how the level of awareness in many clinical conditions can vary regarding the perspective in which the information is presented. For example, in the context of anosognosia for hemiplegia, Ramachandran and Rogers-Ramachandran (1996) reported that subjects may appraise the paralysis of others while denying any motor deficit in themselves, as well as both Fotopoulou et al. (2009) and Besharati et al. (2015) showed that self-observation through videos could improve awareness of their own motor deficits. Videos with self-performance also enhanced awareness of psychotic episodes in psychotic individuals (Davidoff et al., 1998; Vikram et al., 2008). PwAD with anosognosia, for instance, have been described to inaccurately estimate their functioning (Cosentino et al., 2007; Mograbi et al., 2012b) in a 1PP. Also, further evidence suggests that despite the compromised ability to monitor and regulate one’s acts (Mograbi et al., 2014), PwAD might preserve their capacity to perceive someone else’s deficits (Bertrand et al., 2016). For instance, Duke et al. (2002) showed that PwAD overestimated their performances while making accurate evaluations of their spouses (Duke et al., 2002). Also, in another study, they were also able to appreciate the deficits of a fictional person, pose suitable advice, and distinguish those from subjects with no sight of impairments when confronted with information through vignettes (Clare et al., 2012a).

It is important to consider that the manipulation of perspectives through experimental procedures would facilitate PwAD to assume an external perspective, a process that perhaps they could not achieve naturally. In any case, the ability to take a 3PP, within self-or other-frameworks, might involve other processes beyond ToM, therefore facilitating the shift in perspective-taking. It is evident the need to better explore the complexity of perspective-taking influence on perceiving and reasoning of performances and deficits among PwAD.

## 4 Tools to assess awareness of condition, symptoms, or cognitive ability

Different models and approaches have been proposed to better investigate aspects of awareness of deficits and the related effects of perspective-taking. However, there is still no consensual methodology or “gold standard” (Ecklund-Johnson and Torres, 2005). In addition, with awareness being described as a complex and multidimensional concept (Clare et al., 2005; Sunderaraman and Cosentino, 2017), these methods mostly tried to measure particular aspects of the phenomenon. There is a lack of assessment methods that evaluate self-awareness in AD through a multidimensional perspective (e.g., Dourado et al., 2014), in particular those that assess awareness of condition, symptoms or cognitive ability.

A recent systematic review examined the availability of methods used to assess, in clinical settings, awareness of the diagnosis or related changes in functioning among PwD (Alexander et al., 2021). Notably, nearly half of the articles centered on a particular object of awareness. Cognition, typically memory function, was the most assessed object, representing 80% of articles. Following, functional ability, measured through basic and/or instrumental ADL, and behavioral domains, mostly social and emotional functioning, were assessed in around 40% of the studies. Awareness of physical symptoms and awareness of the diagnosis of dementia was also observed to a lesser extent (Alexander et al., 2021). For clinical applications, the direct relevance of a particular impairment in patients’ life should be considered. Despite cognitive deficits being highly prevalent in PwAD, functional deficits, as in ADLs, cause a high impact on both their’ and their carers’ daily life. For instance, the lack of awareness of this type of difficulty might result in dangerous behavior and the delay of related diagnosis, which may be relevant to determine potential clinical and research focus (Gauthier et al., 2021). Also, loss of awareness can vary among PwAD and many times, more than one object of awareness can be impaired (Kotler-Cope and Camp, 1995), which should be valued to reason approaches examining more than one object of awareness.

According to this recent review, quantitative methods used to measure awareness can be broadly categorized into (1) questionnaires eliciting discrepancies between self-and informant-ratings, such as the “Anosognosia questionnaire-Dementia” (Migliorelli et al., 1995); (2) discrepancies between self-ratings and objective measurement of performance, for instance, the “Memory Awareness Rating Scale” (Clare et al., 2005); and (3) brief clinical ratings based on responses to an interview, as the Reed Anosognosia Rating Scale (Reed et al., 1993). Authors highlighted how self-rating approaches in PwD could have reduced reliability, as highly influenced by the personality, self-concept, and mood of the patients (Clare et al., 2012a,b; Martyr et al., 2019; Alexander et al., 2021), as well as by the unconscious or preconscious process of denial (Clare et al., 2012b). Furthermore, they argued how the self-expression of one’s own experiences would be a fundamental part of the subjective experience of awareness, therefore, crucial to portray the phenomenon as a whole (Clare, 2003; Mardh et al., 2013; Alexander et al., 2021).

Clare et al. (2011) investigated awareness in early-stage dementia by applying cluster analysis techniques within a multidimensional approach. The authors discussed how multidimensional assessments could indeed offer a more accurate examination of subjects’ level of awareness, as well as explore possible predictors and correlates (Clare et al., 2011). Nevertheless, there is still a lack of a standard method that explores the plurality of this concept and fills the overlap between the difficulties in both cognitive functions and ADL (Martyr and Clare, 2012; Alexander et al., 2021), as well as investigates the potential influences of perspective-taking on awareness of a condition or cognitive and behavioral changes.

In this framework, vignettes address the issues of personality and self-concept effects on the recognition and interpretation of impairments in other subjects with dementia (Alexander et al., 2021), being mostly employed in face-to-face interviews, videotapes, audiotapes, and computers (for a review, Torres, 2009). These are hypothetical scenarios regarding situations of a fictional character that permit participants to explore the circumstances described with detachment from them (Torres, 2009; Clare et al., 2012a). Participants are asked subsequent questions about the character’s deficits and potential medical problems in a way of informing about their understanding of the scenario (Torres, 2009) while avoiding the occurrence of an impression-management bias (Alexander and Becker, 1978) and the pressure of socially desirable answers (Torres, 2009). This approach activates mental representations of cognitive impairments, behaving as a stimulus for the patient to evaluate the deficits related to their disease (Alexander and Becker, 1978) and consequently, as a way for clinicians and researchers to assess their ability to appraise them (Schmand et al., 1999; VonDras, 2009). Clare et al. (2012a) argued that individuals with AD, vascular dementia, or mixed AD and vascular dementia were able to distinguish dementia from healthy aging vignettes and were less prone to use terms such as “Alzheimer’s” or dementia and more adapted to general descriptions of the situation. The authors discussed that problem identification and response might involve distinct processes and even with conscious avoidance or unconscious denial, these individuals could still identify signs of dementia in others with the use of vignettes (Clare et al., 2012a). In addition, even with no requirement of a comparison between the hypothetical story and the self, self-reference processes commonly occur (Clare et al., 2012a) and offer valuable insights into how PwD might perceive their symptoms.

It is important to note, however, this method represents only frames of experiences and the responses prompted through them do not necessarily match how participants would perceive the related situations (Schultheis et al., 2002; Torres, 2009). In that way, vignettes may not operate as the most appropriate tool to infer potential behavior (Torres, 2009). Also, the use of vignettes may place high cognitive demands on the participants. Within an experimental task, to listen to stories, to appraise them, and to imagine how one would behave accordingly, might represent a cognitive challenge, therefore compromising a part of the analysis, especially in cases of individuals with cognitive impairments. Moreover, each vignette will most likely discuss a theme regarding one particular object of awareness. Consequently, for a study to perform a more global assessment, it might need to introduce a considerable number of stories, which would demand a high capacity of attention and focus, often compromised among PwD. Therefore, there is still the need for a more realistic and perhaps person-centered approach that appraises with accuracy the great range of deficits in AD regarding awareness of condition and ability.

## 5 Virtual reality

VR can be defined as advanced computer interfaces (Rizzo et al., 2020) that simulate three-dimensional images or environments (Diniz Bernardo et al., 2021) with different degrees of complexity and enabling the integration of real-time computer graphics, body tracking devices, visual displays, and other sensory input. The computer-based simulation permits to explore and manipulate the scenario (Diniz Bernardo et al., 2021), and test cognitive and sensorimotor skills (Fuchs et al., 2006; Plancher et al., 2010; Tuena et al., 2019, 2020; Kruse et al., 2022). VR allows immersive experiences in a safe environment, while controlling the stimuli involved and their level of intensity (Diniz Bernardo et al., 2021). Its use in neuropsychology has been growing in recent years, as it allows researchers and clinicians to simulate daily life conditions in experimental naturalistic environments, involving subjects in cognitive and sensorimotor tasks that might function as individualized evaluations (Plancher et al., 2010; Valladares-Rodriguez et al., 2017; Strong, 2020). With a high ecological validity, it allows participants to be involved in a series of real-world settings, such as cities, supermarkets, and domestic activities, thus enabling the appraisal of their complex abilities and actual functioning (Muratore et al., 2019).

This technological tool might present variances in the level of complexity and interaction, allowing the user to experience immersion at different intensities. For instance, Muratore et al. (2019) categorized VR devices into non-immersive, immersive, and semi-immersive systems, mainly depending on the equipment and technology used (D’Cunha et al., 2019). The first, the simplest method, relates to virtual environments created mostly through a desktop. The second, such as head-mounted displays, isolate users from the outside world and provide broader body ownership and sensory interactions with a 360° immersive virtual scenario. The last employs an illusion of technological non-mediation, thus offering a greater sense of reality (D’Cunha et al., 2019; Muratore et al., 2019). VR tools, independently of their immersion levels, typically involve decision-making and learning processes (D’Cunha et al., 2019).

Virtual systems may enable the recognition of the individual’s own face and body (Penaud et al., 2022) and provide a sense of embodiment in virtual avatars (Kilteni et al., 2012; Debarba et al., 2015). The immersive experience encompasses the users’ visual field, adapting itself correspondingly to their orientation and physical movements (Kim et al., 2019; Clay et al., 2020) to such a degree that would hardly be possible in reality with people with neurocognitive disorders, such as dementia. Indeed, both the feeling of presence (Kilteni et al., 2012; Cipresso et al., 2018; La Corte et al., 2019) and the self-recognition evolved through the control of self-movement (Tsakiris et al., 2005), as well as the use of personalized avatars (Iriye and St. Jacques, 2021; Penaud et al., 2022) are necessary for PwAD to behave according to their actual abilities. In that way, this technology might address the challenge of examining some of the main features of AD, and could be classified according to: (1) intended purpose (e.g., assessment and diagnosis; cognitive training or therapy; and caregivers’ training), (2) impairment characteristics it is focused on (e.g., memory deficit), (3) methodology employed, (4) kind of virtual environment, and (5) type of interaction technique (e.g., level of immersion and passive or active interaction etc.) (García-Betances et al., 2015).

Recent systematic reviews have discussed how these immersive virtual environments could positively impact both examination and training of cognitive and psychological functioning in dementia population, as well as their diagnosis (D’Cunha et al., 2019; Moreno et al., 2019; Kruse et al., 2022; Buele et al., 2023). García-Betances et al. (2015) also indicated how having this technology in PwD homes and residential aged care communities could provide them with greater overall accessibility. It is important to note that the authors discuss how most reported implementations of VR tools among PwAD have occurred within low-level immersive VR rather than immersive VR (García-Betances et al., 2015).

In the same vein, Strong (2020) review discussed how both immersive and non-immersive technologies are already being used in studies with PwD and how specific VR equipment might allow these analyses to more accurate and feasible insights with this population. The authors also comment on the tolerance and acceptance of PwD to engage with the immersive virtual environment and the avatars, in a way of proposing VR as a safe and agreeable tool for both treatment and research purpose (Strong, 2020). In this line, Appel et al. (2014) investigated the acceptability of VR among PwD, analyzing the total time of participants viewing VR, aspects of comfort (reported levels of pain, weight and other discomfort), and adequacy of stimuli (whether there were sufficient visual and auditory stimuli). Safeness was also explored through the correlates of patient nervousness or anxiety, confusion or disorientation, or the occurrence of any interaction with a medical device. Despite some reports of anxiety or discomfort (mainly due to the weight of the headset), the study suggested VR as an accepted and safe treatment tool for PwD (Appel et al., 2014). On the other hand, some studies report skepticism and concerns from family members and caregivers regarding the use of VR among PwD on possible mental confusion, negative emotions, fearfulness, and risk of falling related to the experience (Rose et al., 2018, 2021; Flynn et al., 2022). Other studies discuss the perceived level of complexity and difficulty (e.g., using the mouse or joystick, or reading virtual signposts) as well as the overall enjoyment in VR testing, which could, in turn, reduce the quality of performance (Cherniack, 2015; Rose et al., 2018 Zhu et al., 2021). Still, the literature also emphasizes how a broader understanding with corresponding hardware and software adjustments, as well as the dosage and type of tasks that best suit PwD can most likely mitigate these negative points related to the use of VR and thus, make this tool even more appropriate for this specific population (Rose et al., 2018; Zhu et al., 2021; Flynn et al., 2022).

Throughout tasks concerning navigation and orientation, cognitive functionality, and ADL, it displays extensive adaptability to the array of characteristics and needs of PwAD (Baus and Bouchard, 2014; García-Betances et al., 2015). Based on this, some studies explored the feasibility of virtual tasks involving episodic memory (Plancher et al., 2012), everyday memory or action-planning daily activities (i.e., supermarket and breakfast preparation) for the assessment of PwAD functional difficulties, and the potential of reaching a diagnosis (Bialystok et al., 2008; Werner et al., 2009; Plancher et al., 2012).These findings might suggest VR as an interesting tool for the assessment of functional limitations in PwAD and perhaps enabling to investigate their ability to appraise and interpret their deficits and/or diagnosis.

## 6 VR: a new tool to explore anosognosia?

**Figure 1:**
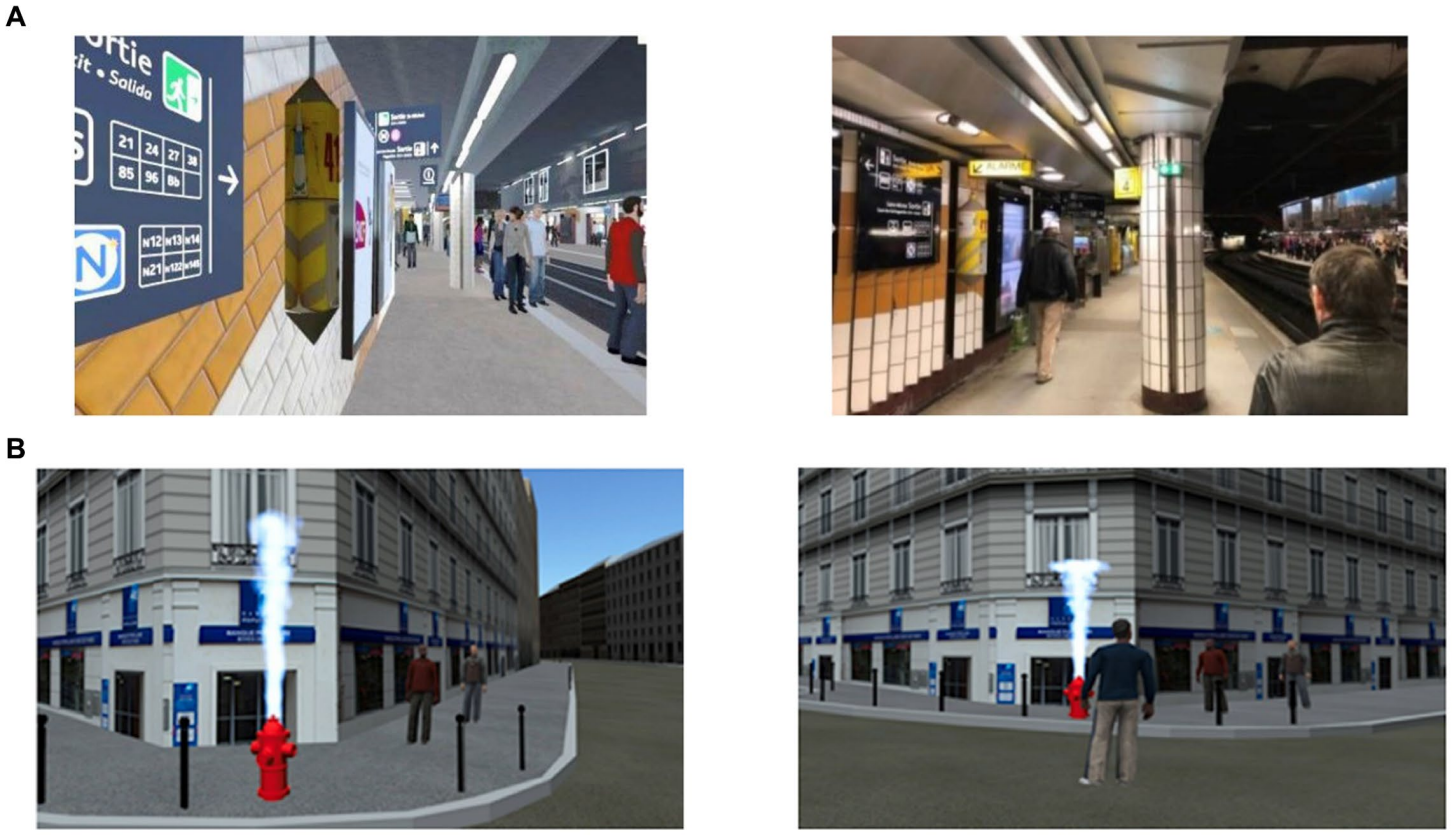
**(A)** Examples of a train station as a virtual environment compared to the real-life environment. **(B)** Examples of a virtual environment in first-person perspective and in third-person perspective (Source: MC2Lab-University Paris Cité).

The great level of complexity, veracity (Salamin et al., 2010; Debarba et al., 2015), and safety (García-Betances et al., 2015) of virtual systems permit to bind participant’s functional and cognitive impairments that are relevant to everyday life in an ecological and naturalistic setting (Widmann et al., 2012; Jebara et al., 2014; Abichou et al., 2019; Penaud et al., 2022). Indeed, VR has been correlated to greater accuracy in memory appraisal-related investigations in comparison to standard methods, notably in aging, mostly due to three central features: first perspective-taking experience, active exploration of the environment, and adaptable scenarios with different degrees of complexity (Plancher et al., 2010, 2012; Repetto et al., 2016; La Corte et al., 2019; Tuena et al., 2019, 2020). In contrast, traditional memory tests often lack dynamic and detailed environments, leading participants to recall primarily static stimuli (Rizzo et al., 2020). VR surpasses these limitations by offering immersive experiences that allow for comprehensive evaluations, including 360-degree self-awareness assessments. For example, individuals can engage in scenarios within a simulated realistic environment (see Figure 1 for examples of realist virtual environments), facilitating a multi-dimensional evaluation of behavior. This approach provides a more holistic and interactive assessment compared to conventional methods.

Also, virtual-based analyses might offer reliable means to experience different situations through both 1PP and 3PP (Iriye and St. Jacques, 2021) and across egocentric and allocentric viewpoints (Weniger et al., 2009). The management of the viewpoint in a virtual environment can be approached through various methods, mostly stemming from the camera’s position within the space. For example, when adopting a 1PP, the subject and the avatar would share the same vision, with the viewpoint at the level of the avatar’s eye. In a 3PP setting, the camera viewpoint would accompany the avatar from behind or aside the side, as adopting the perspective of an external observer (Gorisse et al., 2017; Iriye and St. Jacques, 2021; Penaud et al., 2022).

Often, 1PP is applied aiming to project greater accuracy, while a 3PP might be useful for relevant exploration and interaction (Taylor, 2002). Studies on virtual games have discussed how a 1PP may be more likely to induce a dissociation of users and the real world, thus a greater sense of immersion, also allowing their own perception of themselves as having direct action in the virtual activity (Taylor, 2002; Denisova and Cairns, 2015; Armougum et al., 2019). With the high degree of immersion and interaction in a 1PP, and the sense of ownership of the virtual body [i.e., one’s self-attribution of a body (Gallagher, 2000)], a sense of bodily self-awareness emerges and head to an enhanced virtual presence (Kilteni et al., 2012; Gorisse et al., 2017; Kim et al., 2019; Penaud et al., 2022). In the case of an egocentric visual of the virtual body, the virtual environment reacts to users as if they were in there, augmenting the sense of self-localization and therefore, self-presence (Lee, 2004). In this sense, self-presence is described as the variation of a behavior or emotional state perceived by the real self through the incorporation of a virtual avatar, which in turn, could increase user’s sense of identity (Lee, 2004; Kilteni et al., 2012).

Egocentric visual representations have been linked to the parietal area and dorsal striatum (Burgess, 2008; Mizumori et al., 2009; Chersi and Burgess, 2015; Tuena et al., 2020), while the processing and maintaining an allocentric reference frame has been associated with activity in the hippocampus, parahippocampal, and retrosplenial cortex (O’Keefe and Dostrovsky, 1971; Wolbers and Büchel, 2005; Zhang and Ekstrom, 2013; Tuena et al., 2020). Bilateral fronto-temporal regions have been suggested as a common network between both allocentric and egocentric frames (Buckner and Carroll, 2007; Zaehle et al., 2007; Cona and Scarpazza, 2019).

In this line, a recent study used a virtual reality navigation task and showed that amnestic MCI and mild AD exhibit more impaired egocentric recall (virtual maze) than allocentric memory retrieval (virtual neighborhood) (Mohammadi et al., 2018). Other VR studies examined a specific dysfunction in the encoding and storing of allocentric representations, with subsequent deficient translation into egocentric ones (Serino et al., 2014, 2015). This finding was later discussed by Rizzo et al. (2020), suggesting VR as a means to achieve neuroplasticity and neural reorganization in AD and consequently, improve their performances (Rizzo et al., 2020). Still, most virtual-based literature focus on the influence of perspective-taking on spatial memory (Montana et al., 2019; Cimadevilla et al., 2023) and episodic memory (Bergouignan et al., 2014; Penaud et al., 2022), lacking studies that directly evaluate the effects of perspective-taking on awareness of a condition or deficits in PwAD.

To our knowledge, Koenig et al. (2011) is one of the few studies to discuss the issue of participants’ awareness of deficits using a VR task. The study examined working memory and the ability to imagine different angular perspectives in a task where participants with brain injury had to memorize the initial position of a target object (for 2 min or less) and move them immediately back to their initial location after they were changed (objects always moved to new locations and viewpoints could remain unchanged, shift 90° or shift 180°). After each session, feedback was given to the patient. Although self-awareness appraisal was not the aim of the analysis, authors suggested a strong effect of the high ecological task on improving awareness of their cognitive deficits for a few of the participants (5 out of 45) (Koenig et al., 2011).

Pavone et al. (2016) also reported relevant data regarding perspective-taking and awareness of error in a study with an immersive VR system and electroencephalogram recording. Participants were immersed in a virtual scenario of a dining room with two conditions: 1PP (avatar’s arm projected out of their shoulder) and 3PP (observation of avatar movements), while EEG signals were recorded. Participants observed one of the avatars performing movements of reaching and grabbing one of two mugs in two different blocks, with a randomized sequence of correct and incorrect trials. The error-related negativity and theta oscillations, the neuroelectric signatures of detecting errors, were associated during the observation of erroneous movement through an active 1PP and were reported only for early and possibly automatic markers of error detection. Moreover, error positivity was comparable in 1PP and 3PP, suggesting that late conscious awareness of errors could be similar for self and others. Authors hypothesized that through a virtual environment, from a passive observation with a 3PP, early markers of error detection would be prompted by the unconscious attribution of salience to self-errors, while late markers of error awareness would be related to a detached coding of errors in both self and others (Pavone et al., 2016).

Despite the scarcity of studies that directly investigate the influence of perspective-taking on self-awareness through VR systems, the existing literature offers interesting grounds for developing new experimental methodologies with this particular purpose, in particular awareness of condition, symptoms or cognitive ability. Such tasks could be based on everyday life environments, allowing a wider range of cognitive abilities to be tested and map real-world behavior. For example, the Jansari assessment of Executive Functions (JEF^©^) creates a virtual environment with everyday multitasking in which participants take on the role of an office worker whose objective is to organize and prepare a meeting (Jansari et al., 2014). Virtual tasks as this enable the assessment of a great range of executive and cognitive abilities, with the possibility of real-time feedback, and a manipulation of subject’s perspective. In the same line, Allain et al. (2014) proposed a virtual kitchen, with highly realistic features (e.g., shelves and cabinet drawers, hob, oven, water noise, etc.), in which participants were instructed to prepare a cup of coffee with milk and sugar. The authors investigated different functional and cognitive aspects, such as the total time to complete the task, the percentage of task steps, the total number of errors, emotion, memory deficits. Tasks like this offer great grounds for everyday activities in which the perspective from which the participant performs the activity could be easily manipulated (Allain et al., 2014). Also, Jebara et al. (2014) proposed a virtual car drive experience in a paradigm in which young and older adults would undergo four distinct conditions: passive, itinerary control, low control, and high control. This study explored the scores in episodic memory obtained throughout the conditions, comparing both active and passive navigation in terms of motor interaction (Jebara et al., 2014).

Previously established immersive techniques that integrate VR real-time computer graphics, visual displays, and other sensory inputs may allow the manipulation of the PwAD perspective to be made more realistically, thus permitting an enriched self-evaluation. The control of some VR features, as the body representation in a way of the level of structure, morphology, and size similitude (Kilteni et al., 2012), as well as the real-time motion capture system that allows to mimic the user’s movement (Gorisse et al., 2017) might contribute to the accuracy in the manipulation of the user’s perspective. It is important to detangle the components of the virtual system as to augment the sense of (1) self-location, (2) of agency, and (3) of ownership in a way to maneuver the potential of the tool and task performance, increasing the reliability of the clinician/research analysis. Virtual tasks would be able to objectively record and quantify the PwAD performance, and subsequently compare self-evaluation and actual performance (Muratore et al., 2019). Overall, VR-based technology seems a promising alternative to compensate for the low ecological validity of classical tests, also offering a useful mechanism for training of self-awareness of cognitive and behavior and quantifiable ecological assessment of deficit awareness.

## 7 Concluding remarks

To summarize, anosognosia can be thought of as a complex and heterogeneous concept, dependent on the inter-and intra-individual variability of profiles of awareness in AD. In this framework, previous findings suggested that PwAD may show better awareness when appraising and judging self-or others-abilities in a 3PP related to a 1PP. However, most methods have focused on unidimensional analysis, failing to assess possible predictors and correlates, and reducing the accuracy in which awareness is examined. Also, they mostly lack ecological and immersive experiences, hampering the investigation of the potential influences of perspective-taking on anosognosia in AD.

By contrast, VR-based methods display a great level of control over existing variables, enabling the development of settings that are adaptable to the diverse characteristics and needs of each specific individual. The technology permits to manipulate the perspective in which users are presented to a scenario, allowing them to actively experience and handle a situation or observe it through an external point of view. In this way, VR might be a safe and effective tool for the accurate assessment of self-awareness and the possible influences of perspective-taking, enabling participants to evaluate their own performance while still experiencing it. Also, these technology-based environments might permit mimicking a range of complex activities, comprising multidimensional assessments of PwAD level of awareness. Previous VR studies might offer sufficient grounds for developing new methodologies with the particular purpose of exploring perspective-taking in awareness of a condition or symptoms or cognitive abilities in AD.

Despite its potential, there are a number of issues with VR that need to be considered before its use. For instance, the existing literature contrasting active and passive interaction in virtual environments has been showing incoherent results on performance (Tuena et al., 2019; Rizzo et al., 2020). The difficulties in performing qualitative and quantitative comparisons (Jebara et al., 2014) might be on account of the divergences between the VR systems (e.g., non-immersive, semi-immersive, and fully immersive simulations), experimental designs, and the measures employed (e.g., interference of verbal distractors, type of participants’ responses to encoded information) (Chrastil and Warren, 2012). Also, the different levels of ecological validity and the variation in the use of sensorimotor stimulation, with its potential effects on cognition may lead to discrepancies between studies that would hamper a reliable comparison (Rizzo et al., 2020).

It is also important to consider that the 1PP and 3PP may behave as independent variables and their related manipulation in virtual tasks might need to be assessed and quantified through separate scales (Iriye and St. Jacques, 2021). Previous findings suggest that perspective-taking is not a binary phenomenon and it could be argued that a particular perspective may be more or less advantageous for memory performances and self-appraisal depending on specific variables. Furthermore, research on perspective-taking in AD has generally focused on spatial cognition, mainly assessing memory encode and recall in a spatial context (Bohil et al., 2011; García-Betances et al., 2015; Serino et al., 2015), and on protocols for cognitive training and for the detection of the disease. That said, there is a considerable lack of consistent data on self-evaluation and awareness of errors in performances through VR. There is still the need to better explore the potential of VR tools concerning perspective-taking shifts in AD, with particular attention to awareness of deficits, a hallmark of the disease.

Clinically, 3PP could be used as a method to reduce the impacts of anosognosia in AD. Within this approach, VR could be a reliable tool for health professionals to investigate individual deficits and possibly improve awareness of PwAD regarding their symptoms and conditions. Notably, the specialists involved in related practices should be responsive to the potential psychological and emotional effects of enhancing individuals’ awareness of deficits and diagnosis. Some other point that should be considered when introducing VR tools older people is the higher probability of problems associated with cybersickness and motion sickness (Strong, 2020), as well as perceived level of difficulty and complexity (Rose et al., 2021 Zhu et al., 2021; Flynn et al., 2022) which could limit the ecological validity of the studies. Also, there is still no evidence of possible differences in the use of immersive VR in community-dwelling versus institutionalized PwD and how this could affect their perception of the activities performed, nor in people with advanced-stage dementia (Strong, 2020). We believe that the prevalence, as well as the impact, of anosognosia in AD justifies the use of VR and perspective-taking as a way to better comprehend the processes involved in self-awareness, potentially having a wide value socially.

## Author contributions

SL-T: Conceptualization, Data curation, Formal analysis, Investigation, Resources, Visualization, Writing – original draft. EB: Formal analysis, Validation, Visualization, Writing – original draft. PP: Formal analysis, Writing – review & editing. DM: Conceptualization, Data curation, Funding acquisition, Investigation, Resources, Supervision, Visualization, Writing – original draft.
